# Supplementary material for: Tangled Up in Fibers: How a Multidomain Lytic Polysaccharide Monooxygenase Binds Its Chitin Substrate
Source: ACS Appl Mater Interfaces. 2026 Feb 19;18(8):13228–41. doi: 10.1021/acsami.5c24418 (PMC12964348; doi:10.1021/acsami.5c24418)
Supplement: Supplementary file 1 [file am5c24418_si_001.pdf]

# SUPPORTING INFORMATION

# Tangled up in fibers: How a multi-domain lytic polysaccharide monooxygenase binds its chitin substrate

Henrik Vinther Sørensen[1,2*], Mateu Montserrat-Canals[1,3], Ayla Coder[1], Sylvain Prévost[4], Susan Krueger[5,6], Gustav Vaaje-Kolstad[7], Kaare Bjerregaard-Andersen[1,§], Reidar Lund[1], Ute Krengel[1*]

[1] Department of Chemistry, University of Oslo, NO-0315 Oslo, Norway

[2] Department of Biomedical Science, Malmö University, SE-205 06, Sweden

[3] Centre for Molecular Medicine Norway, University of Oslo, NO-0318 Oslo, Norway

[4] Large-Scale Structures group, Institut Laue-Langevin, 71 avenue des Martyrs, 38042 Grenoble, France

[5] Department of Materials Science and Engineering, University of Maryland, College Park, Maryland 20742, United States

[6] Center for Neutron Research, National Institute of Standard and Technology, Gaithersburg, Maryland 20899, United States

[7] Faculty of Chemistry, Biotechnology and Food Science, Norwegian University of Life Sciences (NMBU), NO-1340 Ås, Norway

[§] Present address: Ottilia vej 9, H. Lundbeck A/S, DK-2500 Valby, Denmark

*Correspondence: Henrik V. Sørensen (henrik.vinther-sorensen@mau.se); Ute Krengel (ute.krengel@kjemi.uio.no)

**ASSOCIATED CONTENT:**

Figure S1: SANS experiments carried out to obtain chitin match point

Figure S2: SANS data of chitin–D-GbpA complex at 73% $D_2O$

Figure S3: Extended TEM micrographs of chitin fibers

Figure S4: Extended TEM micrographs of chitin–GbpA complexes

Figure S5: TEM micrographs with differing concentration ratios of chitin and $GbpA_{fl}$

Figure S6: Extended TEM micrographs of chitin fibers incubated with $GbpA_{D1-3}$

Figure S7: Extended TEM micrographs of chitin fibers incubated with $GbpA_{D1}$

Table S1: SANS fitting parameters for chitin cylinder model with power law

Table S2: $GbpA_{fl}$ : chitin ratios in the dilution-series TEM experiment

Table S3: $GbpA_{fl}$ : chitin ratios in the direct-mixing TEM experiment

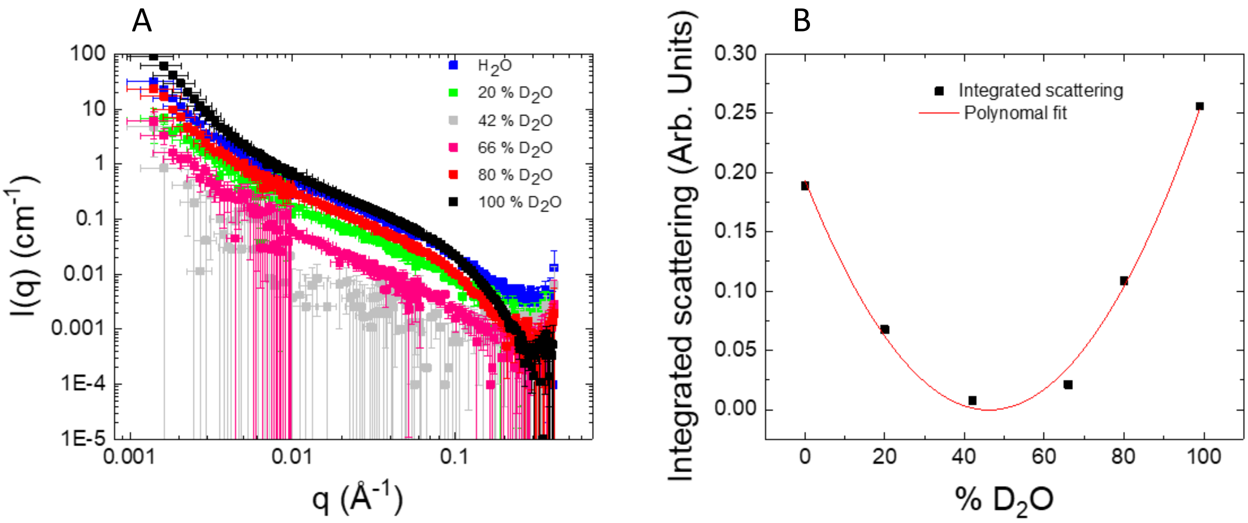


**Figure S1 SANS experiments carried out to obtain chitin match point. A** 10 mg/mL chitin was subjected to SANS at six different ratios of $D_2O$ to $H_2O$, and the data were plotted as intensities (I(q)) vs. the scattering vector (q) on double-logarithmic scale. **B** The scattering for each of the six conditions was integrated, plotted and fitted with a polynomial function, to find the chitin match point at minimal scattering intensity (determined to be at 47% $D_2O$).

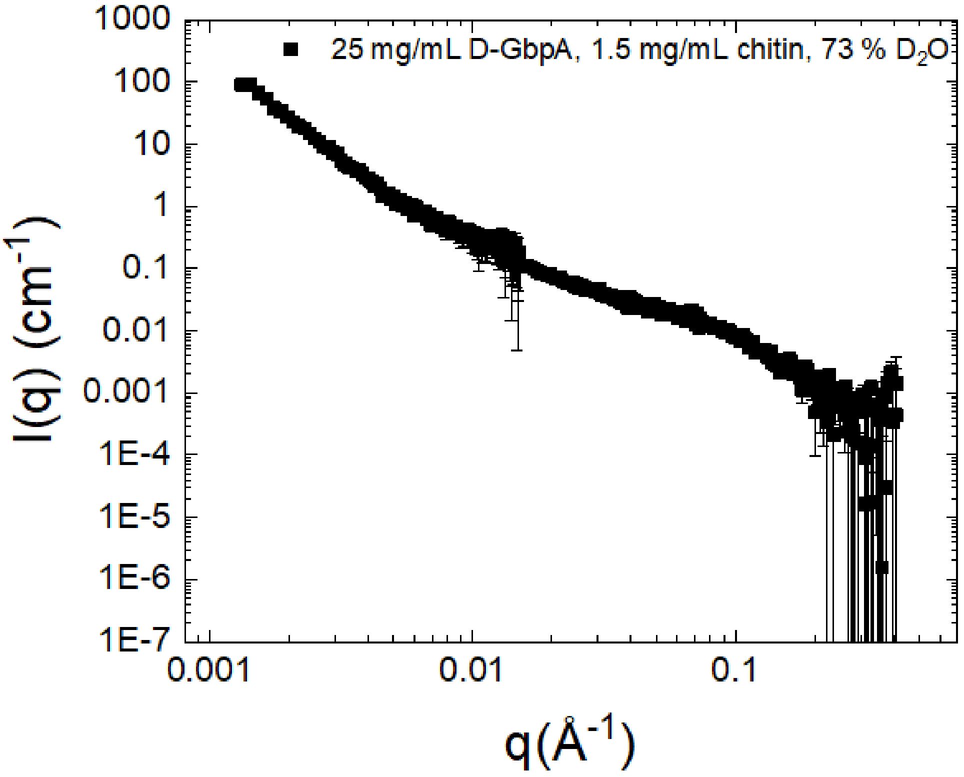


**Figure S2 SANS data of chitin–D-GbpA complex at 73% $D_2O$.** Measurement in the middle of the chitin and D-GbpA match point yielded a slope close to the average of the slope for chitin and the complex at 47% $D_2O$. Error bars represent one standard deviation.

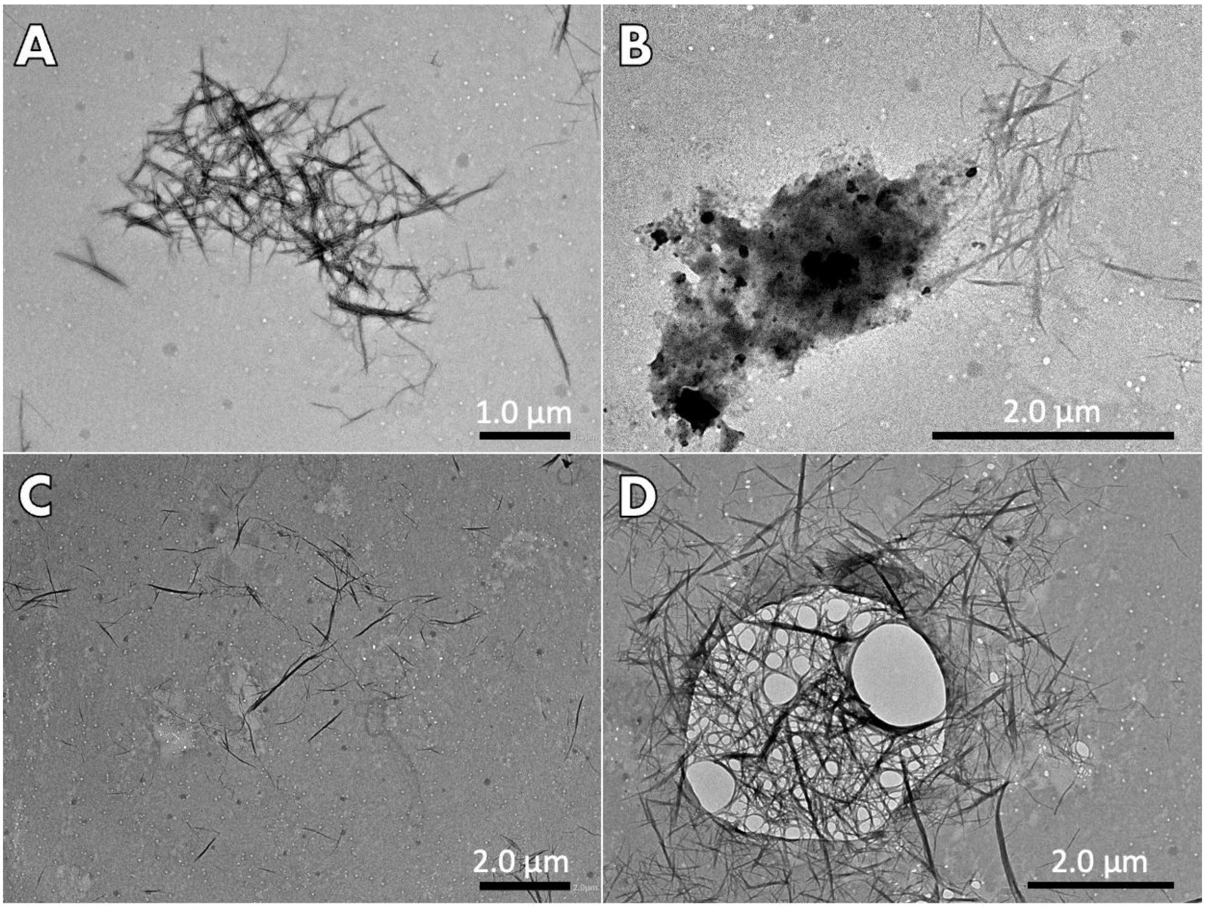


**Figure S3 Extended TEM micrographs of chitin fibers**. **A** A cluster of short chitin fibers. **B** An unknown contamination or crystallization next to a cluster of short chitin fibers. **C** Several chitin fibers spread out more evenly. **D** A cluster of chitin fibers that have been trapped in a hole in the formvar film. The concentration of chitin in these samples was 1.5 mg/mL.

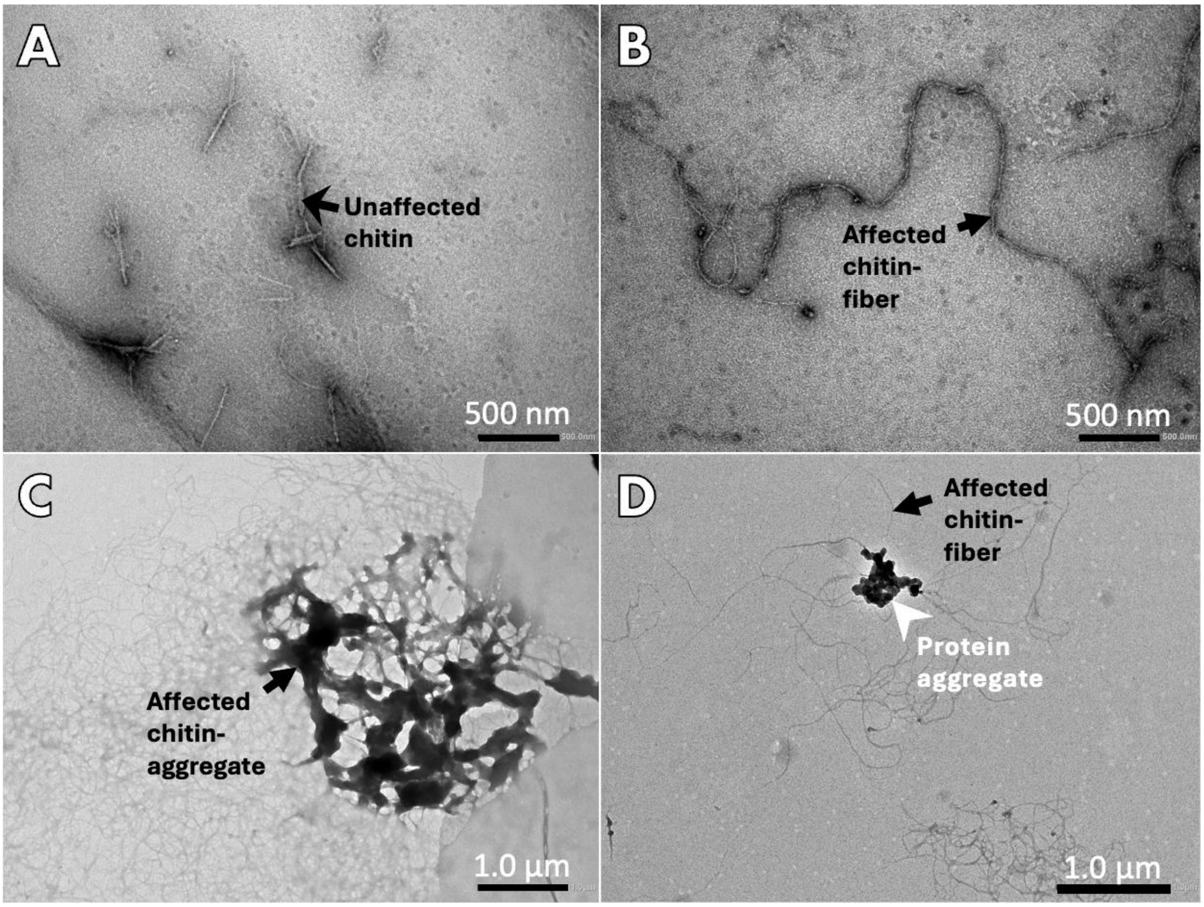


**Figure S4 Extended TEM micrographs of chitin–GbpA complexes**. **A** A few relatively unaffected chitin fibers were present in this sample. **B** Close-up image of elongated fibers created by $GbpA_{fl}$, presumably coating (and potentially linking) the chitin fibers. Note the smoother appearance compared to uncoated fibers (Figure S3). **C** Image of one of the many dense aggregates of chitin observed after complex formation with $GbpA_{fl}$. From the cluster, a plethora of elongated fibers spread out from this denser aggregation, representing "classic" $GbpA_{fl}$-coated chitin fibers shown in B. The hard line at the right side of this image is presumably an artifact. **D** Another GbpA-chitin cluster that is ultrastructurally characteristic of a protein aggregate, with several elongated chitin-$GbpA_{fl}$ fibers extending from it. Additional elongated fibers are found at the bottom of the image. The concentration of chitin in these samples was 1.5 mg/mL, while the concentration of $GbpA_{fl}$ was 400 $\mu$M.

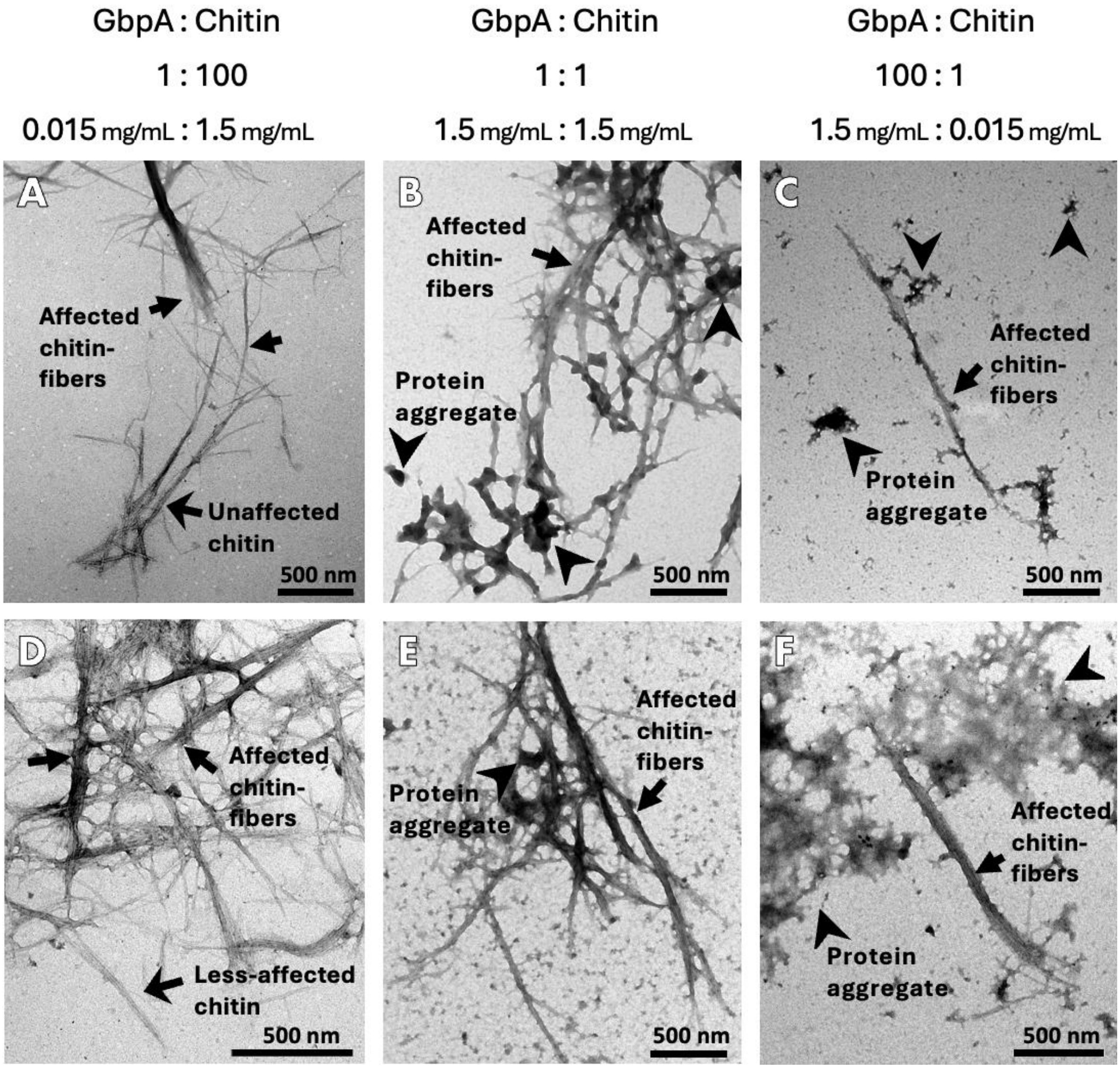


**Figure S5 Extended TEM micrographs with differing concentration ratios of chitin and GbpA$_{fl}$.** (**A-C**) Shows representative results from the serial dilution experiment, while (**D-F**) are representative of results from the samples that were individually prepared, without serial dilutions. These different sample preparations had an inrteresting effect on the results. **A** In this sample containing 0.015 mg/mL GbpA: 1.5 mg/mL chitin, completely unaffected chitin fibers (broad arrow) were observed right next to GbpA$_{fl}$-coated chitin fibers (slim arrows). Comparing this to the 1.5 mg/mL GbpA: 1.5 mg/mL chitin sample in **B**, no unaffected chitin was observed. Additionally, more numerous and denser aggregates of protein (tail-less arrows) were observed on the GbpA$_{fl}$-coated chitin. **C** Less large clusters of chitin-fibers like were found in this sample containing 1.5 mg/mL GbpA: 0.015 mg/mL chitin (in some micrographs, one large cluster dominated the image; not shown). Most of the grid was covered in very small, dense protein aggregates that were not associated with any chitin fibers. The few chitin fibers that were found were all coated with GbpA$_{fl}$. Essentially, this image shows a 100-fold dilution of B, with the addition of some small protein aggregates. **D** Like in A, the sample contained 0.015 mg/mL GbpA: 1.5 mg/mL chitin (however, in this case prepared directly, without prior serial dilution). No unaffected chitin fibers with sharp edges were found. **E** This sample was prepared as in B, and thus represents a replicate of B. **F** Sample comparable to C, but prepared directly, without prior serial dilution. The results are similar to C, except for the size and quantity of the protein-aggregates. The differences between **A** and **D**, but not between **B-C** and **E-F** are likely a result of the speed and affinity (with low off-rates) at which GbpA$_{fl}$ binds to chitin. In the dilution experiment, it appears that when GbpA$_{fl}$ and chitin were mixed in the 1:1 sample (**B**), the protein bound to the chitin so fast and with such high affinity that upon dilution, the coverage on the already-affected chitin changed very little and the added chitin remained uncoated by GbpA.

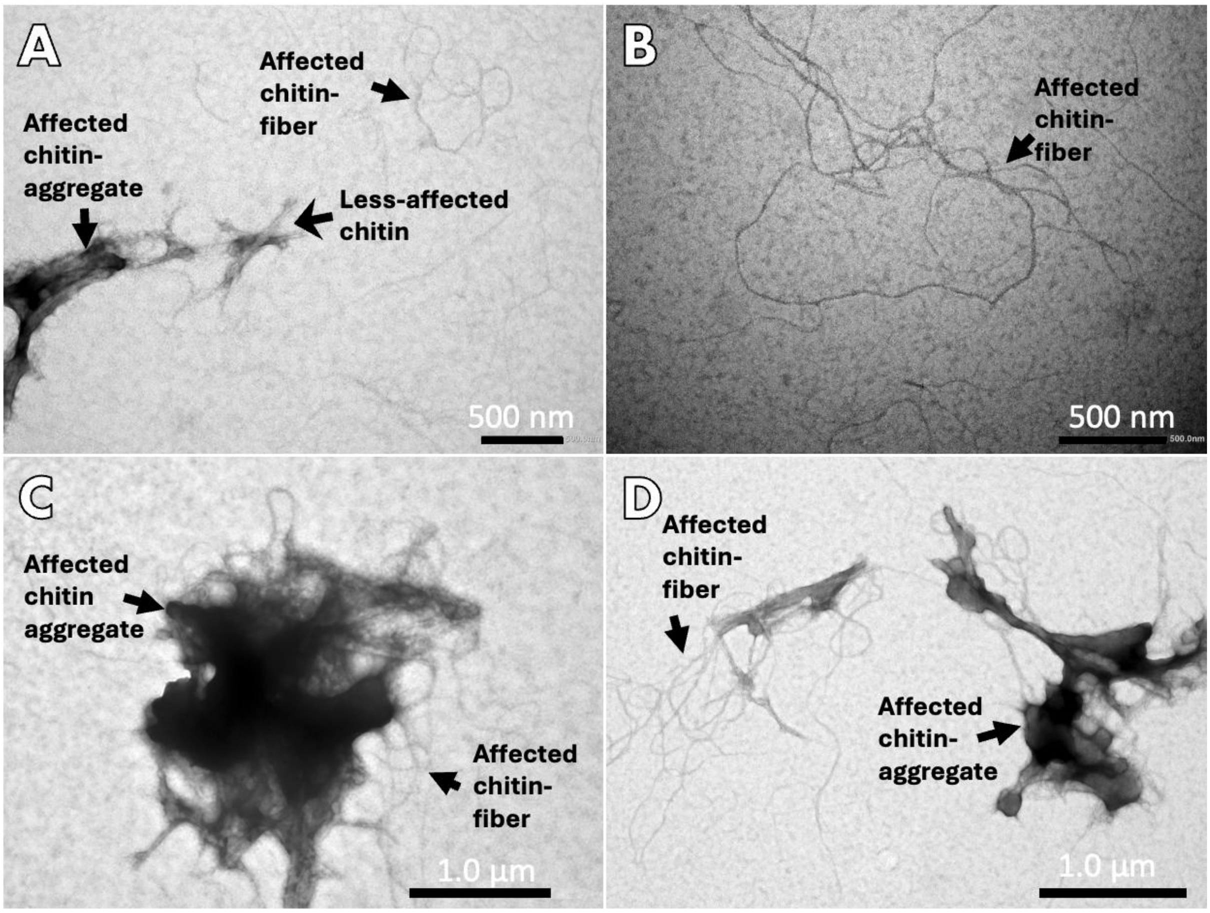


**Figure S6 Extended TEM micrographs of chitin fibers incubated with GbpA$_{D1-3}$. A** A cluster of aggregated chitin affected by GbpA$_{D1-3}$ is visible on the left, with sharper, more defined chitin fibers right next to it in the center of the image. Classic, individual, elongated looping fibers of chitin affected by GbpA$_{D1-3}$ are also present. **B** Close-up view of individual chitin fibers coated with GbpA$_{D1-3}$. **C** Cluster of GbpA$_{D1-3}$-chitin aggregate with surrounding individual fibers. **D** Two smaller clusters of GbpA$_{D1-3}$- chitin aggregates with individual fibers extending from them. The concentration of chitin in these samples was 1.5 mg/mL, while the concentration of GbpA$_{fl}$ was 400 µM.

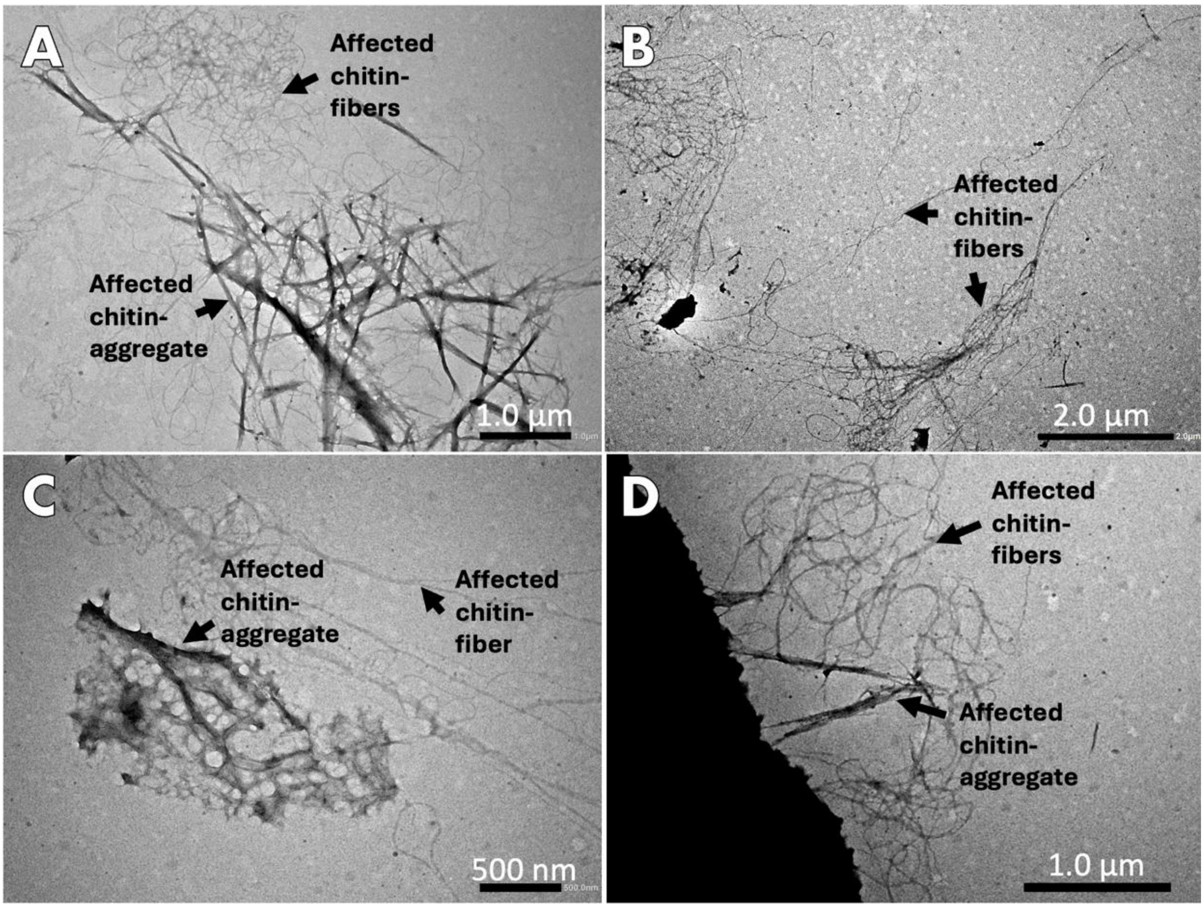


**Figure S7 Extended TEM micrographs of chitin fibers incubated with GbpA$_{D1}$. A** Cluster of aggregated chitin fibers incubated with GbpA$_{D1}$, with individual fiber loops extending above from the aggregate. Compared to chitin complexes with GbpA$_{fl}$ (Figure S4) or GbpA$_{D1-3}$ (Figure S5), the chitin fibers appear sharper and more defined. **B** Close-up view at individual GbpA$_{D1}$-coated chitin fibers. **C** Cluster of GbpA$_{D1}$-chitin aggregate with surrounding individual fibers. **D** Two smaller clusters of GbpA$_{D1}$-chitin aggregates with individual fibers extending from them. The aggregates clearly have more jagged edges than for the longer GbpA constructs (Figures S4-5). The concentration of chitin in these samples was 1.5 mg/mL, while the concentration of GbpA$_{fl}$ was 400 $\mu$M.

**Table S1: SANS fitting parameters for chitin cylinder model with power law**

| Sample | Length (Å) | Kuhn length (Å) | Cylinder radius (Å) | Decay exponent | $\chi^2$ | SASBDB ID |
|---|---|---|---|---|---|---|
| Chitin (3 mg/mL), 100% $D_2O$ | 5000 | 318 +/- 12 | 15.99 +/- 0.02* | 3.99 +/- 0.01 | 2.78 | SASDW42 |

* *Note that the statistical error from the modeling is likely much lower than the actual uncertainty of the value.*

**Table S2*:* GbpA$_{fl}$ : chitin ratios in the dilution-series TEM experiment**

| Sample (GbpA:chitin) | 1:1000 | 1:100 | 1:10 | 1:1 | 10:1 | 100:1 | 1000:1 |
|---|---|---|---|---|---|---|---|
| GbpA$_{fl}$ (mg/mL) | 0.0015 | 0.015 | 0.15 | 1.5 | 1.5 | 1.5 | 1.5 |
| Chitin (mg/mL) | 1.5 | 1.5 | 1.5 | 1.5 | 0.15 | 0.015 | 0.0015 |

**Table S3*:* GbpA$_{fl}$ : chitin ratios in the direct-mixing TEM experiment**

| Sample (GbpA:chitin) | 1:100 | 1:1 | 100:1 |
|---|---|---|---|
| GbpA$_{fl}$ (mg/mL) | 0.015 | 1.5 | 1.5 |
| Chitin (mg/mL) | 1.5 | 1.5 | 0.015 |
